# Expanding the Diet for DIET: Electron Donors Supporting Direct Interspecies Electron Transfer (DIET) in Defined Co-Cultures

**DOI:** 10.3389/fmicb.2016.00236

**Published:** 2016-03-01

**Authors:** Li-Ying Wang, Kelly P. Nevin, Trevor L. Woodard, Bo-Zhong Mu, Derek R. Lovley

**Affiliations:** ^1^Department of Microbiology, University of Massachusetts, AmherstMA, USA; ^2^State Key Laboratory of Bioreactor Engineering and Institute of Applied Chemistry, East China University of Science and TechnologyShanghai, China; ^3^Shanghai Collaborative Innovation Center for Biomanufacturing TechnologyShanghai, China

**Keywords:** syntrophy, methanogenesis, *Geobacter*, *Methanosaeta*, *Methanosarcina*

## Abstract

Direct interspecies electron transfer (DIET) has been recognized as an alternative to interspecies H_2_ transfer as a mechanism for syntrophic growth, but previous studies on DIET with defined co-cultures have only documented DIET with ethanol as the electron donor in the absence of conductive materials. Co-cultures of *Geobacter metallireducens* and *Geobacter sulfurreducens* metabolized propanol, butanol, propionate, and butyrate with the reduction of fumarate to succinate. *G. metallireducens* utilized each of these substrates whereas only electrons available from DIET supported *G. sulfurreducens* respiration. A co-culture of *G. metallireducens* and a strain of *G. sulfurreducens* that could not metabolize acetate oxidized acetate with fumarate as the electron acceptor, demonstrating that acetate can also be syntrophically metabolized via DIET. A co-culture of *G. metallireducens* and *Methanosaeta harundinacea* previously shown to syntrophically convert ethanol to methane via DIET metabolized propanol or butanol as the sole electron donor, but not propionate or butyrate. The stoichiometric accumulation of propionate or butyrate in the propanol- or butanol-fed cultures demonstrated that *M. harundinaceae* could conserve energy to support growth solely from electrons derived from DIET. Co-cultures of *G. metallireducens* and *Methanosarcina barkeri* could also incompletely metabolize propanol and butanol and did not metabolize propionate or butyrate as sole electron donors. These results expand the range of substrates that are known to be syntrophically metabolized through DIET, but suggest that claims of propionate and butyrate metabolism via DIET in mixed microbial communities warrant further validation.

## Introduction

Direct interspecies electron transfer (DIET) is a possible alternative to interspecies hydrogen transfer for interspecies electron exchange, but the full scope of the environmental significance of DIET has yet to be explored. DIET is possible when microorganisms forge electrical connections, either through biological structures that provide a route for cell-to-cell extracellular electron exchange (Summers et al., 2010; Shrestha et al., 2013a; Rotaru et al., 2014a,b), or via non-biological conductive materials such as magnetite (Kato et al., 2012a,b; Liu et al., 2015), granular activated carbon (Liu et al., 2012; Rotaru et al., 2014a), biochar (Chen et al., 2014b), or carbon cloth (Chen et al., 2014a).

Metatranscriptomic and community composition analysis suggested that DIET was the primary mechanism for interspecies electron exchange in anaerobic digesters converting brewery waste to methane (Morita et al., 2011; Rotaru et al., 2014b; Shrestha et al., 2014). This conclusion was further supported in studies with defined co-cultures. *Geobacter* and *Methanosaeta* species were the predominant bacteria and methanogen species in the digesters (Morita et al., 2011; Rotaru et al., 2014b). Co-cultures of *Geobacter metallireducens* and *Methanosaeta harundinacea* syntrophically metabolized ethanol via DIET (Rotaru et al., 2014b), as did co-cultures of *G. metallireducens* and *Methanosarcina barkeri* (Rotaru et al., 2014a). Although ethanol was the primary substrate in the brewery digesters (Shrestha et al., 2014), ethanol is a minor intermediate in many anaerobic environments where syntrophic metabolism of short-chain fatty acids, such as butyrate and propionate, is likely to account for a greater proportion of carbon and electron flow than syntrophic metabolism of ethanol.

Indirect evidence for the possible metabolism of propionate and butyrate via DIET has come from studies in which magnetite was added to methanogenic communities. Magnetite promotes DIET in defined co-cultures (Kato et al., 2012b; Liu et al., 2015), presumably because it can facilitate electron exchange in a manner similar to that of outer-surface *c*-type cytochromes (Liu et al., 2015). Magnetite amendments promoted the conversion of propionate (Cruz Viggi et al., 2014) and butyrate (Li et al., 2014) to methane and several lines of evidence suggested that this could be attributed to the added magnetite enhancing DIET. Similar magnetite amendment studies have suggested that benzoate (Zhuang et al., 2015) and acetate (Kato et al., 2012a) may also be syntrophically metabolized via DIET under methanogenic conditions.

However, it is difficult to make definitive conclusions about mechanisms for interspecies electron transfer from studies with complex mixed communities in which multiple pathways for electron exchange may be operating simultaneously. Defined syntrophic co-cultures with *G. metallireducens* offer the possibility of conclusively investigating the potential for the metabolism of a diversity of electron donors via DIET because: (1) *G. metallireducens* can grow syntrophically via DIET, but is not capable of interspecies hydrogen or formate transfer (Shrestha et al., 2013b; Rotaru et al., 2014b) and (2) *G. metallireducens* can utilize a wide range of organic electron donors for anaerobic respiration (Lovley et al., 1989, 1993; Lovley and Lonergan, 1990; Sun et al., 2009).

In addition to the methanogens described above, *G. sulfurreducens* has routinely been used as the electron-accepting partner in studies on DIET with *G. metallireducens* because its mechanisms for extracellular electron exchange are better understood than those in methanogens, and because it can be genetically manipulated for functional studies (Summers et al., 2010; Shrestha et al., 2013a,b). Interspecies electron transfer is necessary for growth of *G. metallireducens/G. sulfurreducens* co-cultures in media in which alcohols, or volatile fatty acids larger than acetate serve as the electron donor and fumarate is provided as the sole electron acceptor because *G. metallireducens* can not use fumarate as an electron acceptor and *G. sulfurreducens* can not metabolize alcohols or volatile fatty acids larger than acetate (Lovley et al., 2011).

We report here on the ability of *G. metallireducens* to utilize organic electron donors while growing via DIET with either *G. sulfurreducens* or methanogens as the electron-accepting partner. The results demonstrate that substrates other than ethanol can support DIET-based syntrophy and that electrons derived from DIET can serve as the sole electron source to support methanogen growth.

## Materials and Methods

### Microorganisms, Media, and Growth Conditions

Co-cultures of *G. metallireducens* with either wild-type *G. sulfurreducens* (Summers et al., 2010) or with the previously described (Ueki and Lovley, 2010) citrate synthase-deficient strain of *G. sulfurreducens* (Shrestha et al., 2013a) were obtained initiated from frozen (-75°C) stocks of the co-cultures deposited in our laboratory culture collection during previous studies. As previously described (Summers et al., 2010; Shrestha et al., 2013a), the cultures were routinely grown under strict anaerobic conditions at 30°C in pressure tubes that contained 10 ml of NBF medium, a defined medium with 10 mM ethanol as the electron donor and 40 mM fumarate as the electron acceptor. Co-cultures of *G. metallireducens* and *M. harudinacea* and *G. metallireducens* and *M. barkeri* were routinely maintained, respectively, on NB modified medium and modified 120 medium with 20 mM ethanol as an electron donor and incubated at 37°C as previously described (Rotaru et al., 2014a,b). All electron donors were added from anaerobic sterilized stocks. When noted granular activated carbon (20 g/L) or magnetite nanoparticles (10 mmol/L) were added to the appropriate medium as previously described (Liu et al., 2012, 2015).

### Analytical Techniques

Concentrations of fatty acids (butyrate, propionate, succinate, fumarate, acetate) were measured with high-performance liquid chromatography (HPLC) and concentrations of alcohols (ethanol, propanol and butanol) and methane were measured with gas chromatography (GC) as previously described (Morita et al., 2011; Rotaru et al., 2014a).

### Quantitative PCR Analysis

The abundance of *G. metallireducens* and *G. sulfurreducens* in co-cultures was determined with quantitative PCR with primers specific for the 16S rRNA gene of each strain as previously described (Summers et al., 2010).

## Results and Discussion

### Substrate Range for DIET with Fumarate as the Final Electron Acceptor

**FIGURE 1:**
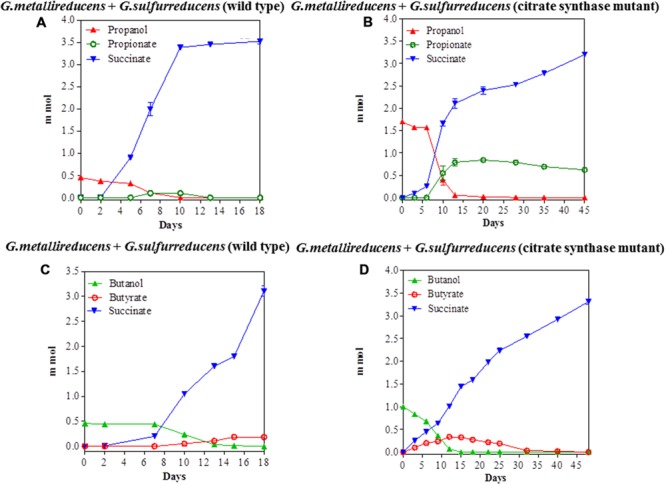
**Syntrophic metabolism in *Geobacter metallireducens/G. sulfurreducens* co-cultures with fumarate as the electron acceptor.** Propanol metabolism with wild type *G. sulfurreducens* **(A)** or the citrate synthase-deficient mutant **(B)** as the electron-accepting partner. Butanol metabolism with wild type *G. sulfurreducens* **(C)** or the citrate synthase-deficient mutant **(D)** as the electron-accepting partner. Each data point represents the mean ± SD of triplicate cultures. In some instances the error bar is smaller than the symbol.

**FIGURE 2:**
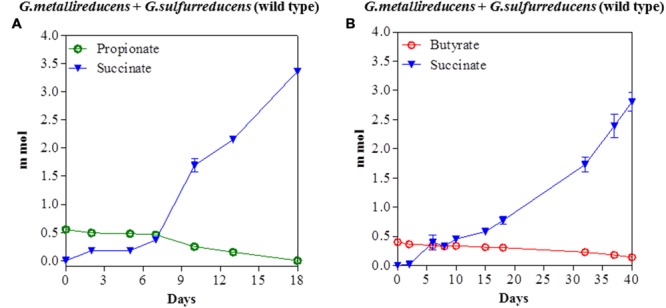
**Syntrophic metabolism of propionate **(A)** or butyrate **(B)** in co-cultures of *G. metallireducens* and wild-type *G. sulfurreducens*.** Each data point represents the mean ± SD of triplicate cultures. In some instances the error bar is smaller than the symbol.

The potential for DIET with substrates other than ethanol was first tested in co-cultures of *G. metallireducens* and *G. sulfurreducens* because this co-culture grows faster than co-cultures of *G. metalllireducens* with methanogens (Rotaru et al., 2014a,b) and the metabolism of the substrates with the reduction of fumarate is more energetically favorable than with the production of methane (**Table 1**). Co-cultures were initiated with the previously described (Ueki and Lovley, 2010) strain of *G. sulfurreducens* which cannot metabolize acetate because the gene for citrate synthase has been deleted, as well as with the wild-type strain. Both types of *G. metallireducens/G. sulfurreducens* co-cultures readily metabolized propanol and butanol with the reduction of fumarate to succinate (**Figure 1**). Propionate or butyrate accumulated, but much less than the 1:1 stoichiometry expected for incomplete oxidation of the alcohols to the respective organic acids (**Table 1**). In some instances (**Figures 1A,D**) the concentration of the organic acids declined with extended incubation, suggesting that they were further metabolized. When either propionate or butyrate were provided as the sole electron donor *G. metallireducens/G. sulfurreducens* co-cultures metabolized these substrates over time (**Figure 2**). The succinate produced (3.36 ± 0.04 mmol; mean ± SD, *n* = 3) from 0.55 ± 0.03 mmol propionate consumed, and 2.8 ± 0.19 mmol succinate produced from 0.28 ± 0.01 mmol butyrate consumed (**Figure 2**) were consistent with the stoichiometry for the oxidation of these substrates with fumarate as the electron acceptor (**Table 1**).

**Table 1:** Standard free energy potentially available at pH 7 from the metabolic reactions investigated calculated with data from (Thauer et al., 1977).

Reactions investigated	ΔG^0′^ (kJ/reaction)^1^
(1) Propanol + H_2_O + 2fumarate^2-^ → 2succinate^2-^+Propionate^-^+H^+^	-159
(2) Butanol + H_2_O + 2fumarate^2-^ → 2succinate^2-^ + Butyrate^-^ + H^+^	-161
(3) Propionate^-^ + H^+^ + 4H_2_O + 7fumarate^2-^→7succinate^2-^ + 3CO_2_	-450
(4) Butyrate^-^ + H^+^ + 6H_2_O + 10fumarate^2-^→10succinate^2-^ + 4CO_2_	-644
(5) Propanol + 5H_2_O + 9fumarate^2-^ →9succinate^2-^ + 3CO_2_	-615
(6) Butanol + 7H_2_O + 12fumarate^2-^ →12 succinate^2-^ + 4CO_2_	-801
(7) Ethanol + 3H_2_O + 6fumarate^2-^ →6succinate^2-^ + 2CO_2_	-425
(8) Acetate^-^ + H^+^ + 2H_2_O + 4fumarate^2-^→4succinate^2-^+ 2CO_2_	-258
(9) Propanol + 1/2CO_2_ → Propionate^-^ + H^+^+ 1/2CH_4_	-58
(10) Butanol + 1/2CO_2_→ Butyrate^-^ + H^+^+ 1/2CH_4_	-53
(11) Propionate^-^+ H^+^+ 1/2H_2_O→7/4CH_4 ↑_+ 5/4CO_2_	-63
(12) Butyrate^-^ + H^+^+ H_2_O→5/2CH_4 ↑_+ 3/2CO_2_	-90

*^1^Reactions for fumarate reduction calculated at 30°C and methane producing reactions calculated at 37°C to reflect the experimental culture conditions.*

Previous studies demonstrated that *Geobacter* species could syntrophically participate in the oxidation of acetate in co-culture with *Wolinella succinogenes* with either nitrate (Cord-Ruwisch et al., 1998; Kaden et al., 2002) or fumarate (Lovley et al., 1999) as the electron acceptor with either a cysteine/cysteine (Kaden et al., 2002) or anthraquione-2,6-disulfonate/anthrahydroquinone-2,6-disulfonate (Lovley et al., 1999) electron shuttle. However, *G. metallireducens* did not appear to substantially metabolize acetate in previous studies on syntrophic metabolism of ethanol via DIET (Shrestha et al., 2013a).

**FIGURE 3:**
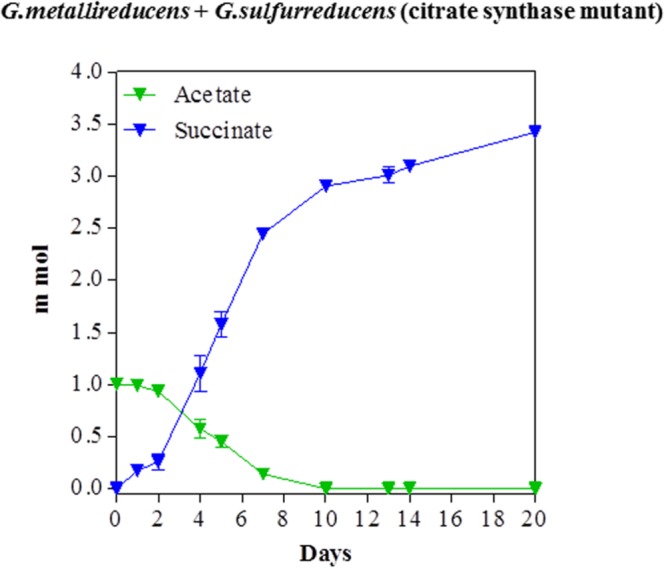
**Syntrophic metabolism of acetate with fumarate as the electron acceptor in co-cultures of *G. metallireducens* and the citrate synthase-deficient of *G. sulfurreducens*.** Each data point represents the mean ± SD of triplicate cultures. In some instances the error bar is smaller than the symbol.

In order to evaluate the potential for syntrophic metabolism of acetate via DIET, acetate was provided as the sole electron donor to co-cultures initiated with the citrate synthase-deficient mutant of *G. sulfurreducens*, to ensure that only *G. metallireducens* would be capable of acetate metabolism. Acetate was consumed over time with a concomitant accumulation of succinate from fumarate reduction (**Figure 3**). Quantitative PCR at the end of the incubation indicated that *G. metallireducens* accounted for 42% of co-culture cells, suggesting that the two *Geobacter* species benefitted almost equally from the electron exchange. The consumption of 1 mmol of acetate was accompanied by the accumulation of nearly 3.4 mmol succinate. This results was in accordance with the stoichiometry of the reduction of four moles of fumarate for each mole of acetate oxidized, (**Table 1**), when the necessity for carbon assimilation for biomass production is considered.

**FIGURE 4:**
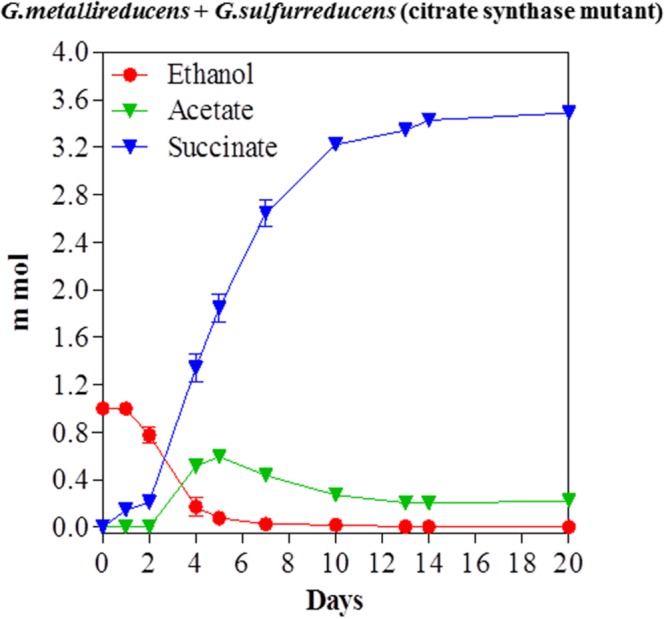
**Syntrophic metabolism of ethanol with fumarate as the electron acceptor in co-cultures of *G. metallireducens* and the citrate synthase-deficient of *G. sulfurreducens*.** Each data point represents the mean ± SD of triplicate cultures. In some instances the error bar is smaller than the symbol.

The previous study on ethanol metabolism in co-cultures of *G. metallireducens* with the citrate synthase-deficient mutant of *G. sulfurreducens* noted nearly stoichiometric production of acetate from ethanol (Shrestha et al., 2013a). However, that study was conducted with 20 mM ethanol, which provides electrons well in excess of the electron-accepting capability of the 40 mM fumarate that was in the medium. When a co-culture of the same strain composition was initiated with 10 mM ethanol, acetate accumulated during ethanol metabolism, but then was subsequently metabolized (**Figure 4**). These results suggest that *G. metallireducens* either preferentially metabolizes ethanol over acetate, or that ethanol metabolism outpaces metabolism of the acetate produced, but that the remaining acetate can be consumed once the ethanol has been depleted.

Monoaromatic compounds can be syntrophically metabolized under methanogenic conditions with interspecies hydrogen transfer (Schink and Stams, 2013; Sieber et al., 2014). Furthermore, cell suspensions of *G. metallireducens* metabolized toluene with *W. succinogenes* as the electron accepting partner and fumarate as the electron acceptor (Meckenstock, 1999) under conditions in which cysteine probably served as an electron shuttle (Kaden et al., 2002). However, attempts to establish *G. metallireducens/G. sulfurreducens* co-cultures with benzoate as the electron donor were unsuccessful even if magnetite or granular activated carbon was added to promote DIET.

### Substrate Range for DIET Coupled to Methane Production

**FIGURE 5:**
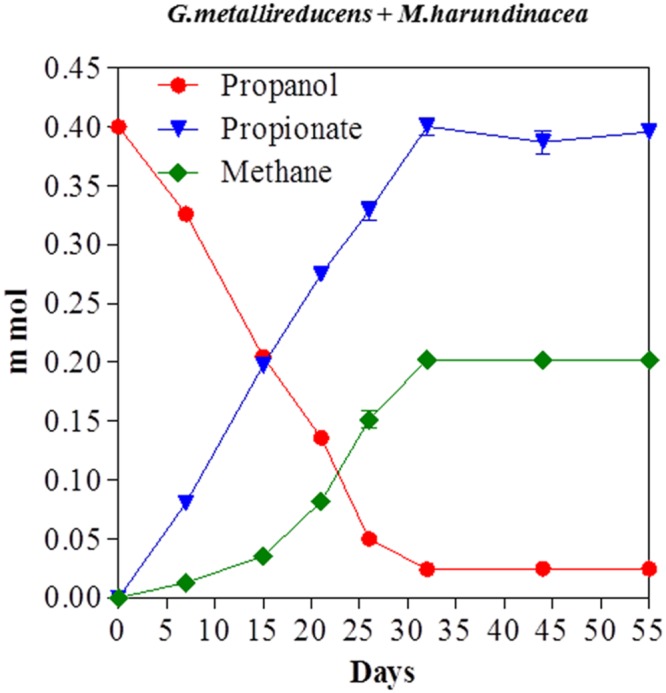
**Propanol consumption with the production of methane and the accumulation of propionate in co-cultures of *G. metallireducens* and *Methanosaeta harundinacea*.** Each data point represents the mean ± SD of quintuplicate cultures. In some instances the error bar is smaller than the symbol.

Propanol was metabolized with the production of methane in co-cultures of *G. metallireducens* and *M. harundinacea* (**Figure 5**). In contrast to the *G. metallireducens/G. sulfurreducens* co-cultures there was a stoichiometric accumulation of propionate in the *G. metallireducens/ M. harundinacea* co-cultures. The amount of methane produced in the propanol co-cultures was 102% of the methane production expected (**Table 1**) for the conversion of propanol to propionate with the electrons derived from the incomplete oxidation of propanol consumed with the reduction of carbon dioxide to methane. Co-cultures of *G. metallireducens* and *M. barkeri* also incompletely metabolized propanol to propionate (Supplementary Material).

**FIGURE 6:**
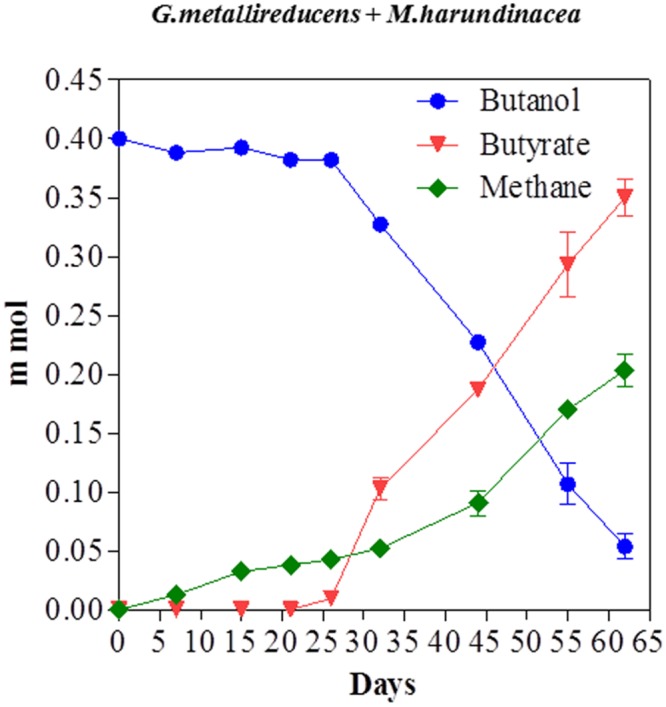
**Butanol consumption with the production of methane and the accumulation of butyrate in co-cultures of *G. metallireducens* and *Methanosaeta harundinacea*.** Each data point represents the mean ± SD of quintuplicate cultures. In some instances the error bar is smaller than the symbol.

Butanol was metabolized in a similar manner with a stoichiometric conversion of butanol to buyrate with 101% of the electrons available from butanol oxidation recovered in methane (**Figure 6**). The successful propagation of these co-cultures on propanol or butanol demonstrated that electrons derived from DIET can serve as the sole source of energy to support the growth of *M. harundinacea*. *G. metallireducens/M. barkeri* co-cultures also produced butyrate from butanol (Supplementary Material).

The accumulation of propionate and butyrate in the *G. metallireducens/M. harundinacea* and *G. metallireducens/M. barkeri* co-cultures suggested that these organisms were not capable of cooperating to metabolize these substrates. Propionate or butyrate added as the sole electron donor to *G. metallireducens/M. harundinacea* co-cultures were not metabolized even when magnetite or granular activated carbon, which promote DIET in co-cultures of *G. metallireducens* (Liu et al., 2012, 2015; Rotaru et al., 2014a), were added to serve as conductive conduits to facilitate interspecies electron transfer (Supplemental Material). Similar results were obtained with *Methanosarcina barkeri* as the methanogenic partner (Supplementary data). In the presence of granular activated carbon the organic acids were removed from solution, which can be attributed to adsorption. When benzoate was added as the sole electron donors it was not metabolized in the *Geobacter*/methanogen co-cultures.

### Implications

These studies substantially increase the range of substrates that are known to be syntrophically metabolized via DIET beyond ethanol and demonstrate for the first time that electrons derived from DIET can serve as the sole energy source to support the growth of a methanogen. Until recently, it was considered that *Methanosaeta* species were only capable of producing methane from acetate (Smith and Ingram-Smith, 2007; Zhu et al., 2012). Thus, growth of *M. harundinacea* under conditions in which the reduction of carbon dioxide to methane was the only mechanism available to support growth further expands its known physiological capabilities.

Although propionate and butyrate were metabolized via DIET in co-cultures in which fumarate was the terminal electron acceptor, attempts to grow co-cultures of *G. metallireducens* and either *M. harundinacea* or *M. barkeri* with propionate or butyrate were unsuccessful, even when magnetite or granular activated carbon were provided to stimulate DIET. One possibility is that metabolic constraints prevent *G. metallireducens* from donating electrons via DIET at a redox potential sufficiently low enough to support methane production when metabolizing propionate or butyrate. However, another possibility is that different culture conditions or longer adaption periods might yield successful co-cultures with *G. metallireducens* and the methanogens with these substrates. In either event, these results emphasize the need for caution when suggesting that methanogenic DIET is possible with propionate and butyrate. In a similar manner, the possibility that magnetite promotes DIET between *Geobacter* and *Methanosarcina* species with acetate as the electron donor (Kato et al., 2012a) should be more directly examined. It seems likely that the hypothesis of DIET in these more complex mixed culture communities could be evaluated with tracer and metatranscriptomic approaches similar to those employed in the study of DIET in brewery waste digesters (Rotaru et al., 2014b).

## Author Contributions

DL and LW designed the experiments. LW cultured the organisms. KN and TW analyzed substrates and metabolites. LW and DL wrote the initial manuscript draft with revisions contributed from all authors.

## Conflict of Interest Statement

The authors declare that the research was conducted in the absence of any commercial or financial relationships that could be construed as a potential conflict of interest.
