# Supplementary material for: Expanding the Diet for DIET: Electron Donors Supporting Direct Interspecies Electron Transfer (DIET) in Defined Co-Cultures
Source: Front Microbiol. 2016 Mar 1;7:236. doi: 10.3389/fmicb.2016.00236 (PMC4772299; doi:10.3389/fmicb.2016.00236)
Supplement: Supplementary file 1 [file Data_Sheet_1.DOCX]

Supplementary Material

Expanding the Diet for DIET: Electron Donors Supporting Direct Interspecies Electron Transfer (DIET) in Defined Co-Cultures

**Li-Ying Wang^1,2^, Kelly P. Nevin^1^,** [**Trevor L. Woodard**](https://www.geobacter.org/trevorwoodard)**^1^, Bo-Zhong Mu^2,3^, Derek R. Lovley^1*^**

*** Correspondence:** Derek Lovley: dlovley@microbio.umass.edu

## Supplementary Figures

**
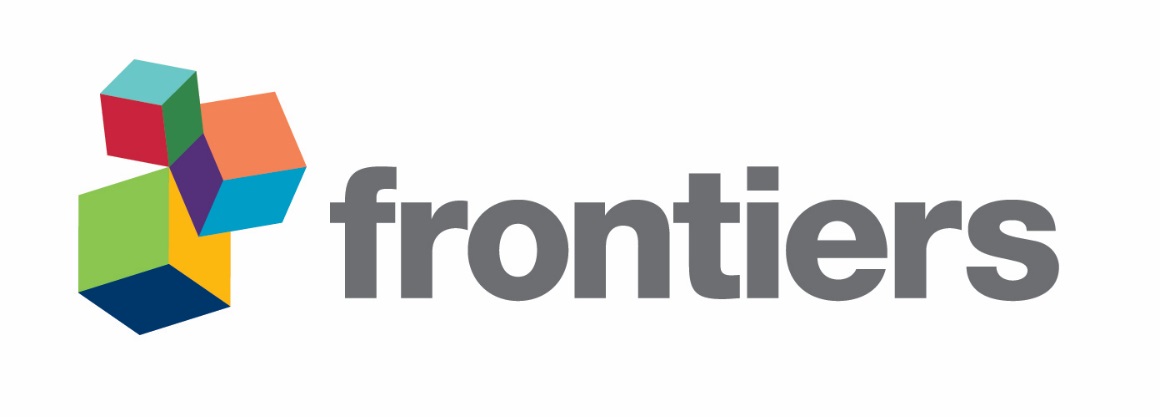
**

**Supplementary Figure 1.** Lack of propionate consumption with or without 20g/L granular activated carbon (GAC) or 10 mmol/L magnetite in co-cultures with *G. metallireducens* and either (A) *Methanosarcina barkeri* or (B) *Methanosaeta harundinacea*. Each data point represents the mean ± standard deviation of triplicate cultures. In some instances the error bar is smaller than the symbol.

**Supplementary Figure 2.** Lack of butyrate consumption with or without 20g/L granular activated carbon (GAC) or 10 mmol/L magnetite in co-cultures with *G. metallireducens* and either (A) *Methanosarcina barkeri* or (B) *Methanosaeta harundinacea*. Each data point represents the mean ± standard deviation of triplicate cultures. In some instances the error bar is smaller than the symbol.

**Supplementary Figure 3.** Propanol consumption with the production of methane production and the accumulation of propionate in co-cultures of *G. metallireducens* and *Methanosarcina barkeri*. Each data point represents the mean ± standard deviation of quintuplicate cultures. In some instances the error bar is smaller than the symbol.

**Supplementary Figure 4.** Butanol consumption with the production of methane production and the accumulation of butyrate in co-cultures of *G. metallireducens* and *Methanosarcina barkeri*. Each data point represents the mean ± standard deviation of quintuplicate cultures. In some instances the error bar is smaller than the symbol.
