# Supplementary figures and images for: Expanding the Diet for DIET: Electron Donors Supporting Direct Interspecies Electron Transfer (DIET) in Defined Co-Cultures
Source: Front Microbiol. 2016 Mar 1;7:236. doi: 10.3389/fmicb.2016.00236 (PMC4772299; doi:10.3389/fmicb.2016.00236)

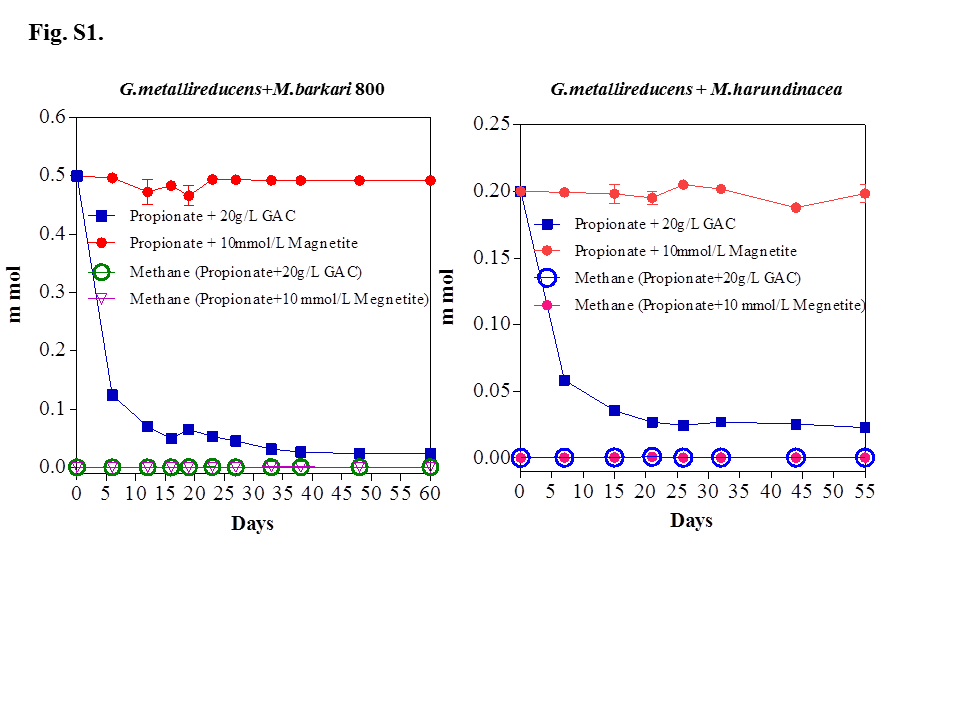

Supplement: Supplementary file 2 [file Image_1.TIF]

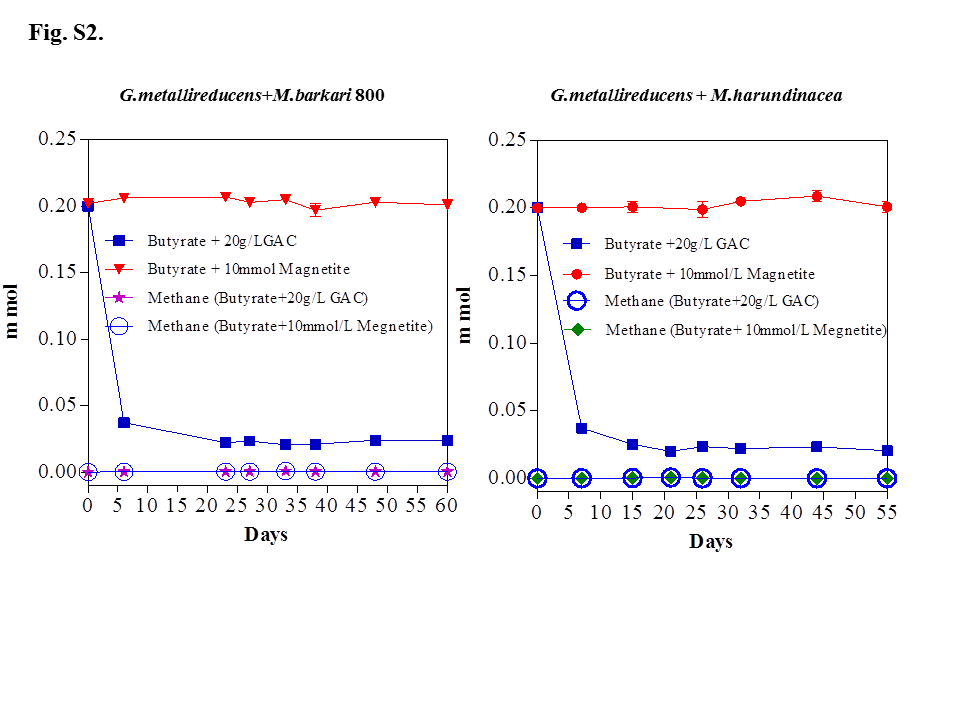

Supplement: Supplementary file 3 [file Image_2.TIF]

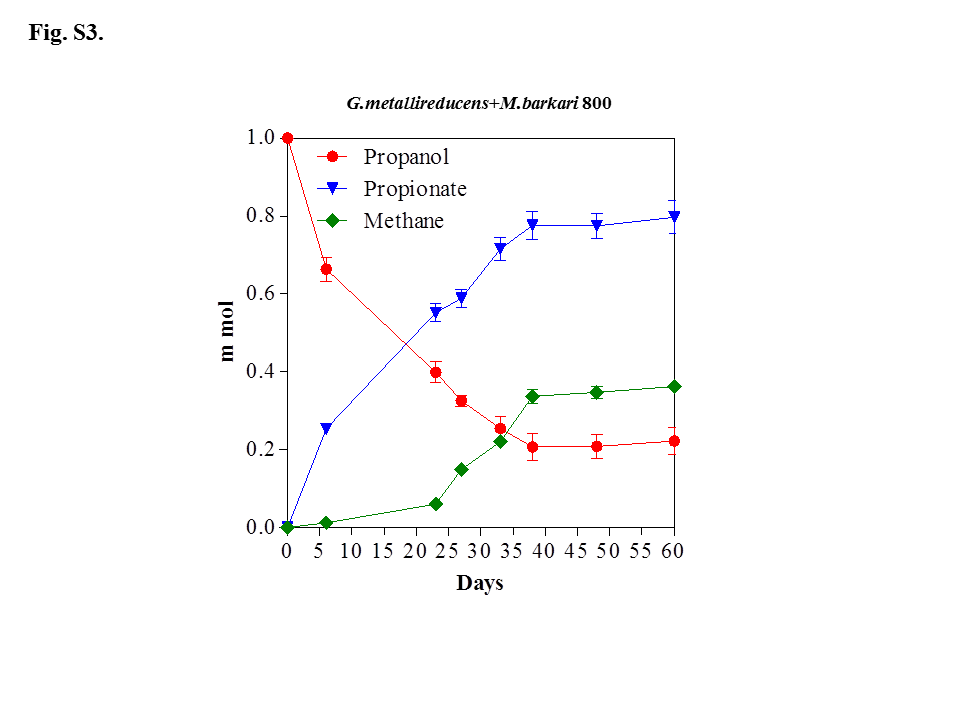

Supplement: Supplementary file 4 [file Image_3.TIF]

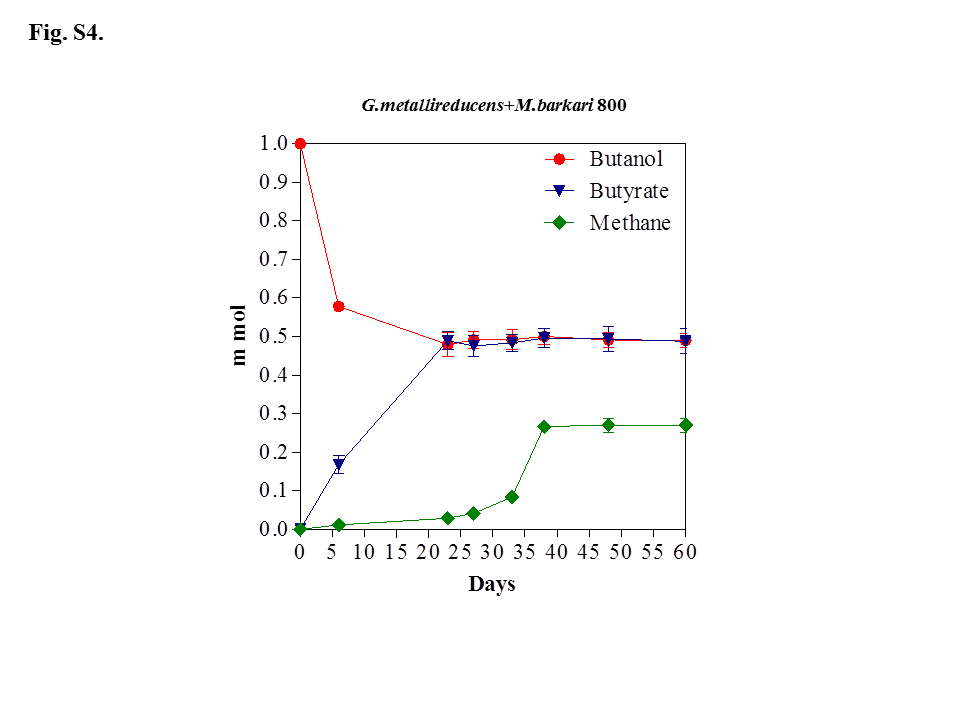

Supplement: Supplementary file 5 [file Image_4.TIF]
